# Prolactin Rescues Immature B-Cells from Apoptosis Induced by B-Cell Receptor Cross-Linking

**DOI:** 10.1155/2016/3219017

**Published:** 2016-05-24

**Authors:** Rocio Flores-Fernández, Francisco Blanco-Favela, Ezequiel M. Fuentes-Pananá, Luis Chávez-Sánchez, Patricia Gorocica-Rosete, Alberto Pizaña-Venegas, Adriana Karina Chávez-Rueda

**Affiliations:** ^1^UIM en Inmunología, Hospital de Pediatría, CMN Siglo XXI, IMSS, 06720 Ciudad de México, DF, Mexico; ^2^Programa de Doctorado en Ciencias Biomédicas, UNAM, 04510 Ciudad de México, DF, Mexico; ^3^Hospital Infantil de México Federico Gómez, Unidad de Investigación en Virología y Cáncer, 06720 Ciudad de México, DF, Mexico; ^4^Departamento de Investigación en Bioquímica, Instituto Nacional de Enfermedades Respiratorias “Ismael Cosió Villegas”, 14080 Ciudad de México, DF, Mexico; ^5^Unidad de Investigación y Bioterio, Instituto Nacional de Enfermedades Respiratorias “Ismael Cosió Villegas”, 14080 Ciudad de México, DF, Mexico

## Abstract

Prolactin has an immunomodulatory effect and has been associated with B-cell-triggered autoimmune diseases, such as systemic lupus erythematosus (SLE). In mice that develop SLE, the PRL receptor is expressed in early bone marrow B-cells, and increased levels of PRL hasten disease manifestations, which are correlated with a reduction in the absolute number of immature B-cells. The aim of this work was to determine the effect of PRL in an* in vitro* system of B-cell tolerance using WEHI-231 cells and immature B-cells from lupus prone MRL/lpr mice. WEHI-231 cells express the long isoform of the PRL receptor, and PRL rescued the cells from cell death by decreasing the apoptosis induced by the cross-linking of the B-cell antigen receptor (BCR) as measured by Annexin V and active caspase-3. This decrease in apoptosis may have been due to the PRL and receptor interaction, which increased the relative expression of antiapoptotic Bcl-xL and decreased the relative expression of proapoptotic Bad. In immature B-cells from MRL/lpr mice, PRL increased the viability and decreased the apoptosis induced by the cross-linking of BCR, which may favor the maturation of self-reactive B-cells and contribute to the onset of disease.

## 1. Introduction

Systemic lupus erythematosus (SLE) is a chronic autoimmune disease that may affect any organ or system in the organism [1, 2]. It is characterized by the presentation of a defect in the tolerance mechanisms (central and peripheral) that give rise to self-reactive T- and B-cell clones, both in patients and in mice that develop SLE [3, 4]. Serum samples from SLE patients characteristically have strong reactivity to a broad spectrum of nuclear components, including DNA, RNA, histones, RNP, Ro, and La. These antibodies form immune complexes that are deposited in the kidneys and may cause proteinuria and kidney failure [5]. SLE is considered a multifactorial disease in which genetic, immunologic, environmental, and hormonal aspects have a close interaction in the development of the disease. SLE incidence is higher in women than in men, and it increases after puberty and decreases after menopause. The severity of SLE also increases during pregnancy [6, 7] and high serum concentrations of PRL correlate with SLE activity [8, 9]. Therefore, the presence of sexual hormones, such as prolactin (PRL), has been associated with this disease [10–12]. In SLE murine models (NZB × NZW and MRL/lpr), the disease activity is exacerbated after induction of hyperprolactinemia, and increased PRL serum levels correlate with the early detection of autoantibodies, proteinuria, and accelerated death [13, 14]. PRL has different functions (over 300) that depend on the type of cell in which its receptor is expressed. There are 4 known PRL isoforms in mice (one long and three short isoforms) [15, 16]. The isoforms present in the extracellular domain are identical, but they differ in size and composition in the intracellular domain. The signaling pathway depends on the isoform that is expressed [17]. Similarly, the PRL receptor is distributed in different cell types, including cells of the immune system [18, 19]. PRL has been implicated as a modulator of both cellular and humoral immunity [20–22].

It has been reported that different maturation stages of B-cells in bone marrow (pro-B, pre-B, and immature) and in the spleen (transitional, marginal zone, and follicular B-cells) express the PRL receptor in mice. However, the expression of the receptor is higher in mice that develop SLE before presenting manifestations of the disease, and the pattern of receptor expression during B-cell development is different in SLE mice from that in mice that do not develop SLE. Additionally, the increase in the PRL serum levels in mice with SLE correlates with a decrease in the absolute numbers of immature and an increase in transitional-1 B-cells, stages that represent important checkpoints for the elimination of self-reactive clones [14, 23].

One of the mechanisms of central tolerance for the elimination of self-reactive clones is clonal deletion, which consists of elimination by apoptosis of immature B-cells that recognize self-antigens with high affinity [24, 25]. To better understand this mechanism, the murine WEHI-231 immature B-cell line has been used as a model to study apoptosis induced by the cross-linking of the B-cell antigen receptor (BCR) [26, 27].

The aim of this work was to determine the effect of PRL in an* in vitro* model of B-cell tolerance. We found that WEHI-231 cells express the long isoform of the PRL receptor and the presence of PRL rescued WEHI-231 cells from apoptosis-mediated cellular death induced by the cross-linking of BCR. The enhanced survival of WEHI-231 cells correlated with increasing the relative expression of antiapoptotic Bcl-xL and decreasing the expression of proapoptotic Bad. In immature B-cells derived from MRL/lpr mice, PRL also increased the viability and decreased apoptosis induced by BCR cross-linking. Taking together our observations in the* in vitro* model of tolerance and in the lupus prone mice, PRL may favor the maturation of self-reactive B-cell clones and contribute to the onset of disease.

## 2. Materials and Methods

### 2.1. Cells

WEHI-231 cells were derived from a B-cell lymphoma in F1 mice (BALB/c × NZB) and were donated by Dr. Leopoldo Santos' laboratory (CINVESTAV, Mexico). The cells were grown in RPMI medium (Hyclone, Utah, USA) supplemented with 10% fetal bovine serum (SFB, Biowest, Riverside, USA), 2-mercaptoethanol (Sigma, St. Louis, USA), and antibiotics (Invitrogen, CAU, USA) at 37°C in 5% CO_2_.

### 2.2. Mice

All studies were approved by the Animal Care Committee of the Instituto Nacional de Enfermedades Respiratorias “Ismael Cosió Villegas” and the Hospital de Pediatría, Centro Médico Nacional Siglo XXI, IMSS (R-2015-785-037), and all the mouse measurements were in accordance with approved guidelines established by Mexico (Norma Oficial Mexicana NOM-062-ZOO-1999) and the NIH* Guide for the Care and Use of Laboratory Animals*. MRL/MpJFAS^lpr^ (MRL/lpr) mice were purchased from the Jackson Laboratory (Maine, USA), and C57BL/6 mice were purchased from Harlan (Indianapolis, USA). Mice were housed in a pathogen-free barrier facility and were provided with sterile food and water* ad libitum*.

### 2.3. Hormones

Recombinant PRL from mice (National Hormone and Peptide Program, NIH) was used.

### 2.4. Antibodies

The following antibodies were used: PECy5-conjugated anti-mouse CD19 (eBio1D3), FITC-conjugated anti-mouse CD43 (eBioR2160), APC-conjugated anti-mouse IgM (11/41), PE-Cy7-conjugated anti-mouse CD23 (B3B4), and PE-conjugated anti-mouse CD93 (AA4.1) antibodies from eBioscience (California, USA); PE-conjugated anti-mouse B220 (RA3-6B2) antibody from BioLegend (California, USA); goat anti-mouse PRL-receptor (PRL-R) (E20) antibody from Santa Cruz Biotechnology (California, CA, USA); and swine anti-goat-biotinylated antibody from Invitrogen (California, USA). The biotinylated secondary antibody was detected with streptavidin-phycoerythrin-Cy5 from eBioscience. The anti-IgM Fab biotinylated and anti-IgM F(ab′)_2_ antibodies were from Jackson ImmunoResearch (Pennsylvania, USA).

### 2.5. Cell Sorting Using WEHI-231 Cells

WEHI-231 cells were incubated with fluorescently labeled antibodies specific for CD43, CD19, IgM, CD23, and goat anti-mouse PRL receptor in staining buffer (PBS with 0.5% BSA) for 20 minutes at 4°C. To select live cells, cells were incubated with DAPI, which marks dead cells (DAPI^+^). The cells were washed and isolated according to the expression of the following surface markers: CD43^−^, CD23^−^, CD19^+^, IgM^+^, PRL receptor^+^, and DAPI^−^ for live cells. Cell sorting was performed using a FACSAria sorter with FACSDiva software (BD Biosciences, California, USA). The purity of the sorted cells ranged from 95% to 98%. For the experiments in which the effect of PRL was tested, cells were cultured in TexMACS medium (Miltenyi Biotec, Bergisch Gladbach, Germany) free of serum, supplemented with 2-mercaptoethanol and antibiotics at 37°C in 5% CO_2_.

### 2.6. Cell Sorting Using Immature B-Cells

Bone marrow (BM) cells were collected by flushing femoral shafts with cold RPMI supplemented with 2% bovine serum albumin (BSA, US Biological, Swampscott, MA, USA) and 2 mM EDTA (IBI Scientific, USA). After depleting red blood cells using lysis buffer (Sigma-Aldrich, St. Louis, Missouri, USA), the cells were incubated with anti-B220 microbeads (Miltenyi Biotec, Bergisch Gladbach, Germany), and the B-cells were isolated using positive selection with a magnetic activated cell-sorting (MACS) system (Miltenyi Biotec, Bergisch Gladbach, Germany). Single-cell suspensions of B220^+^ B-cells from BM were incubated with fluorescently labeled antibodies specific for CD43, B220, IgM, and CD23 in staining buffer (PBS with 0.5% BSA) for 20 minutes at 4°C, and cells were incubated with DAPI to select live cells (DAPI^−^). The cells were washed, and the immature B-cells were isolated according to the expression of the following surface markers: B220^+^, CD43^−^ CD23^−^, IgM^+^, and DAPI^−^. Cell sorting was performed using a FACS Influx Sorter (BD Biosciences). The purity of the sorted cells ranged from 95% to 98%.

### 2.7. Real-Time PCR

Total RNA was extracted from B-cells using TRIzol reagent (Invitrogen, California, USA) according to the manufacturer's protocol, and the RNA concentration was determined using UV spectrophotometry. Total RNA (0.5 *μ*g) was used to generate cDNA with SuperScript II reverse transcriptase (Invitrogen, California, USA) according to the manufacturer's specifications. Genes of interest were amplified by real-time PCR using a LightCycler TaqMan Master kit (Roche Diagnostics, Mannheim, Germany) according to the manufacturer's specifications and using hydrolysis probes and primers designed by Roche Diagnostics. The following primers were used: PRL receptor, 5′-CAGTAAATGCCACGAACGAA-3′ (left) and 5′-GAGGAGGCTCTGGTTCAACA-3′ (right); PRL receptor large, 5′-AGCAGTTCTTCAGACTTGCCCTT-3′ (left) and 5′-AAGCCAGACCATGGATACTGGAG-3′ (right); PRL receptor short, 5′-TTGTATTTGCTTGCAGAGCCAGT-3′ (left) and 5′-AAGCCAGACCATGGATACTGGAG-3′ (right); Bcl-xL, 5′-GCATTGTTCCCGTAGAG-3′ (left) and 5′-GGACCGCGTATCAGAG-3′ (right); Birc5 (survivin), 5′-CCCGATGACAACCCGATA-3′ (left) and 5′-CATCTGCTTGACAGTGAGG-3′ (right); Bad, 5′-GGAGCAACATTCATCAGCAG-3′ (left) and 5′-TACGAACTGTGGCGACTCC-3′ (right); and *β*-actin, 5′-AAGGCCAACCGTGAAAAGAT-3′ (left) and 5′-GTGGTACGACCAGAGGCATAC-3′ (right). The final volume of the reaction was 10 *μ*L, and a LightCycler instrument was used to perform the PCR reaction (Roche Diagnostics). The following PCR conditions were used: 15 minutes at 95°C, followed by 40 cycles of 10 seconds at 95°C, 30 seconds at 60°C, and 1 second at 72°C, and 1 cycle of cooling for 30 seconds at 50°C. The samples were normalized to the *β*-actin gene. The relative expression was calculated using the 2ΔCT formula.

### 2.8. PCR Array

The Mouse CAPM12814F RT^2^ Profiler*™* PCR Array (Qiagen, Hilden, Germany) was performed in 96-well plates following the manufacturer's recommendations using the* RT* ^*2*^ *SYBR Green ROX qPCR Mastermix *(Qiagen). We analyzed 12 genes of interest, 1 constitutive gene, and the following 3 controls to validate each sample: HGDC (DNA genomic control), TRC (retrotranscription efficiency), and PPC (presence of PCR inhibitors). The Mouse PAMM-012ZF RT^2^ Profiler*™* PCR Array (Qiagen, Hilden, Germany) was performed in 96-well plates following the manufacturer's recommendations using the* RT* ^*2*^ *SYBR Green ROX qPCR Mastermix *for apoptosis.

### 2.9. Viability Assay

WEHI-231 cells (PRL receptor^+^) and immature B-cells from MRL/lpr mice were incubated with PRL (50 ng/mL) for 1 hour before stimulating the cells with anti-IgM F(ab′)_2_ (10 *μ*g/mL) for 48 hours and 18 hours, respectively. Cells cultured with medium, PRL, or anti-IgM F(ab′)_2_ were used as controls. The cells were washed with PBS and incubated with Ghost-Red (Tonbo Biosciences, California, USA) for 30 minutes at 4°C; Ghost-Red was used to measure viability (live cells do not stain and remain Ghost-Red^−^). Data were acquired using a MACSQuant Analyzer 10 cytometer (Miltenyi Biotec) and analyzed with FlowJo software (Tree Star, Ashland, OR, USA).

### 2.10. Apoptosis Assays

WEHI-231 cells (PRL receptor^+^) and immature B-cells from MRL/lpr mice were incubated with PRL (50 ng/mL) for 1 hour before stimulating the cells with anti-IgM F(ab′)_2_ (10 *μ*g/mL) for 48 hours and 18 hours, respectively. Cells cultured with medium, PRL, or anti-IgM F(ab′)_2_ were used as controls. The cells were washed with PBS and incubated with Ghost-Red (Tonbo Biosciences) for 30 minutes at 4°C. The Annexin V assay was performed following the manufacturer's instructions (BD Biosciences). For the caspase-3 assay, the cells were permeated with Cytofix/Cytoperm (BD Biosciences) for 1 hour at 4°C, and the cells were then washed with Perm/wash (BD Biosciences) and incubated with anti-caspase-3-FITC for 1 hour at 4°C. Data were acquired using a MACSQuant Analyzer 10 cytometer (Miltenyi Biotec) and analyzed with FlowJo software (Tree Star, Ashland, OR, USA).

### 2.11. Statistical Analysis

Data were analyzed with standard statistical tests (mean value, SD, Student's *t*-test, and ANOVA), and the results are expressed as the means ± SD. The level of significance was set at *p* < 0.05. All calculations were performed using SPSS 22 software.

## 3. Results

### 3.1. PRL Receptor Expression in WEHI-231 Cells

**Figure 1:**
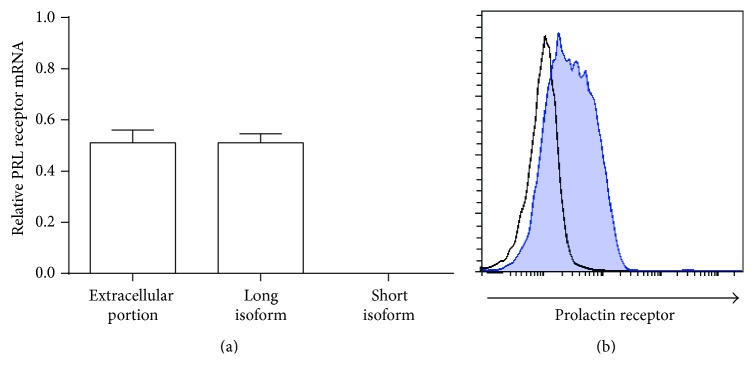
Expression of the PRL receptor. mRNA and protein expression levels of the PRL receptor in WEHI-231 cells. (a) The PRL receptor mRNA expression was measured by RT-PCR using primers specific for the extra- and intracellular domains of the receptor in WEHI-231 cells to allow recognition of the different isoforms (long and short). (b) The expression level of the receptor was measured by flow cytometry using goat anti-PRL receptor antibodies. The isotype control was an unrelated goat antibody.

The expression of the PRL receptor in WEHI-231 cells was determined at both mRNA and protein levels. We first tested whether WEHI-231 cells express the PRL receptor by PCR using primers directed against the extracellular moiety of the receptor, common to all PRL receptor isoforms. After confirming PRL receptor expression (0.51 ± 0.05), primers directed against the intracellular portion of the receptor showed that the WEHI-231 cells only expressed the mRNA of the long isoform (0.51 ± 0.04), as shown in Figure 1(a). With regard to the protein levels, 47.50 ± 5.36% of the cells were positive for expression of the receptor on surface (Figure 1(b)). PRL receptor positive cells were sorted obtaining a 95–98% pure fraction in all cases (Supplementary Figure 1, in Supplementary Material available online at http://dx.doi.org/10.1155/2016/3219017). All experiments were carried out with the PRL receptor positive sorted population grown in RPMI medium supplemented with 10% of FBS, conditions in which PRL receptor positive cells always outgrow PRL receptor negative cells (Supplementary Figure 2). To test for PRL function, PRL receptor positive cells were incubated in TexMACS medium free of serum.

### 3.2. Characterization of WEHI-231 Cells

**Figure 2:**
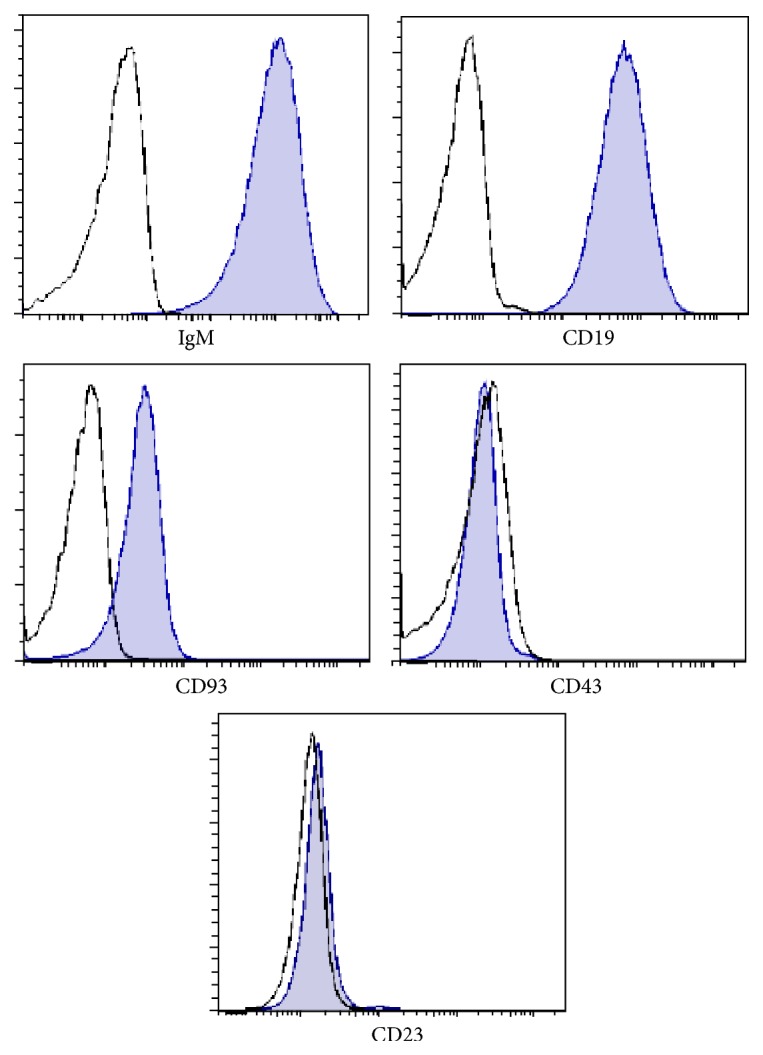
Phenotype of WEHI-231 cells. WEHI-231 cells were stained with Ghost-Red (viability marker) and the following antibodies to determine their phenotype: IgM, CD43, CD23, CD19, and CD93.

**Figure 3:**
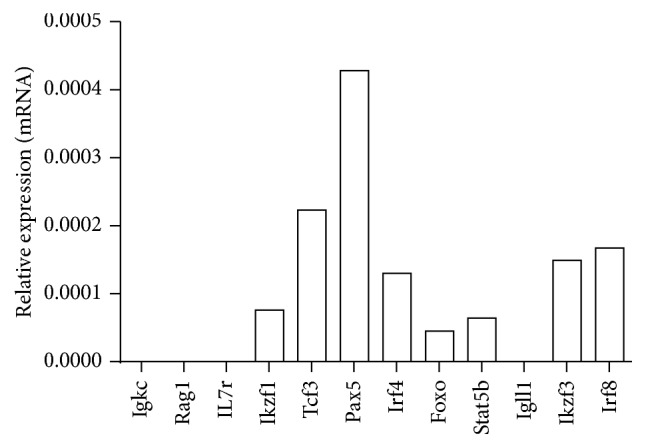
Expression of early development genes in WEHI-231 cells. RNA was extracted from WEHI-231 cells, and cDNA was obtained to determine the expression of genes related to the early development of B lymphocytes by a PCR array.

The phenotype of the WEHI-231 cells was determined by flow cytometry. The WEHI-231 cells were positive for CD93, CD19, and IgM but negative for CD43 and CD23 corresponding to immature bone marrow B-cells (Figure 2). The relative expression of the genes related to the maturation of B-cells was determined by a PCR array, which analyzed the following genes: the constant region of the light kappa chain (lgkc); the Rag1 recombinase; the IL-1 receptor (IL7r): the light subrogated *λ*5 chain (IgII1); and the transcription factors Ikaros (Ikzf1), E2A (Tcf3), Pax5, Irf4, Foxo1, Stat5b, Ailos (Ikzf3), and Irf8. The results showed that WEHI-231 cells do not express genes that are important for the maturation stages of pro- and pre-B-cells, such as IL7r, Rag1, and Igll1, but the results showed that WEHI-231 cells did express Ikzf1 (7.60 × 10^−5^), Tcf3 (2.23 × 10^−4^), Pax5 (4.28 × 10^−4^), Irf4 (1.30 × 10^−4^), Irf8 (1.67 × 10^−4^), Foxo1 (4.50 × 10^−5^), Ikzf3 (1.49 × 10^−4^), and Stat5b (6.40 × 10^−5^), as shown in Figure 3. These data argue that WEHI-231 cells are committed to the B-cell lineage expressing important transcription factors critical for lineage maintenance which are in a post-VDJ rearrangement stage. Igkc was negative implying that these cells express a BCR with lambda light chains.

### 3.3. PRL Effect on Viability and Apoptosis of WEHI-231 Cells

**Figure 4:**
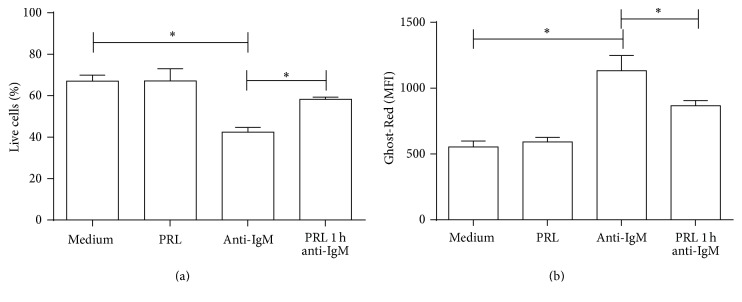
Viability of WEHI-231 cells. WEHI-231 cells were preincubated for 1 hour with PRL and incubated with anti-IgM F(ab′)_2_ antibody for 48 hours. Dead cells were stained with Ghost-Red. (a) Percentage of live cells. (b) Mean intensity of fluorescence (MIF), Ghost-Red. ^*∗*^ *p* < 0.01.

Immature B-cells are constantly being subjected to negative selection mechanisms to check whether their BCRs are directed against self-antigens. To measure how PRL influences the viability and apoptosis outcome of WEHI-231 cells, they were preincubated for 1 hour with PRL and for 48 hours with the anti-IgM F(ab′)_2_ antibody to induce cross-linking of the BCR, a step that mimics self-antigen recognition. The percentage of live and apoptotic cells was measured by flow cytometry. Cells that were incubated with anti-IgM F(ab′)_2_ showed a significantly decreased percentage of live cells (40.93 ± 0.87%; *p* < 0.01) compared to the cells incubated with medium (65.72 ± 1.96%) or PRL (67.10 ± 5.90%). However, cells that were preincubated with PRL and incubated with anti-IgM F(ab′)_2_ showed a significantly increased percentage of live cells (58.42 ± 0.82%; *p* < 0.01) compared to cells that were not preincubated with PRL, as well as a similar percentage of live cells to those incubated only with medium (Figure 4).

**Figure 5:**
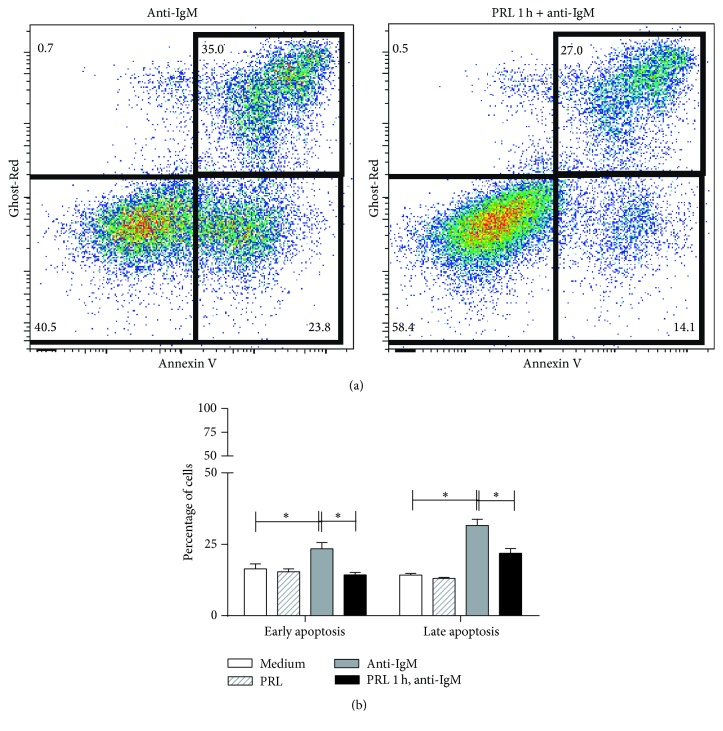
Apoptosis of WEHI-231 cells as measured by Annexin V. WEHI-231 cells were preincubated for 1 hour with PRL and then incubated with an anti-IgM F(ab′)_2_ antibody for 48 hours. The cells were stained with Ghost-Red and Annexin V-FITC to measure early apoptosis (Annexin V^+^ Ghost-Red^−^) and late apoptosis (Annexin V^+^ Ghost-Red^+^). (a) Dot-Plot representative of early and late apoptosis. (b) Percentage of cells in apoptosis. ^*∗*^ *p* < 0.01.

Apoptosis measurement was performed using two different parameters as follows: (1) Annexin V and Ghost-Red staining and (2) active caspase-3. The percentage of cells in early apoptosis (Annexin V^+^ Ghost-Red^−^) and late apoptosis (Annexin V^+^ Ghost-Red^+^) significantly increased (23.88 ± 2.56 and 31.62 ± 2.66%; *p* < 0.01) for cells incubated with anti-IgM F(ab′)_2_ compared to cells incubated with medium (16.23 ± 2.02 and 14.28 ± 0.71%) or PRL (15.37 ± 0.97 and 13.0 ± 0.44%). A significant decrease (14.44 ± 0.99 and 21.92 ± 2.00%; *p* < 0.01) was found in early and late apoptosis for cells preincubated with PRL and incubated with anti-IgM F(ab′)_2_ as compared to cells not preincubated with PRL (Figure 5).

**Figure 6:**
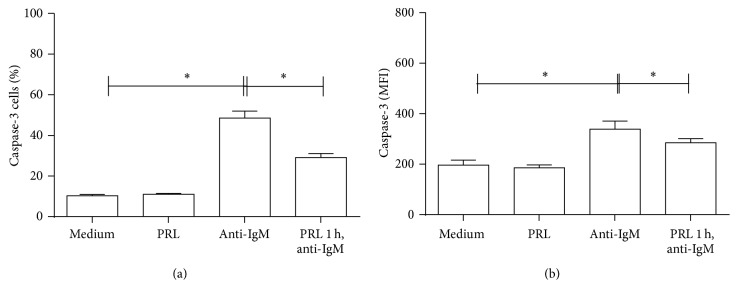
Apoptosis of WEHI-231 cells as measured by active caspase-3. WEHI-231 cells were preincubated for 1 hour with PRL and incubated with anti-IgM F(ab′)_2_ antibody for 48 hours. The cells were stained with Ghost-Red and active caspase-3-FITC to determine apoptosis. (a) Percentage of caspase-3+ cells. (b) IMF of caspase-3. ^*∗*^ *p* < 0.01.

In contrast, the percentage of cells with active caspase-3 significantly increased (50.76 ± 1.35%; *p* < 0.01) for cells incubated with anti-IgM F(ab′)_2_ as compared to cells incubated with medium (9.99 ± 0.33%) or PRL (11.0 ± 0.44%). The percentage of cells with active caspase-3 significantly decreased (29.50 ± 1.93%; *p* < 0.01) for cells preincubated with PRL and incubated with anti-IgM F(ab′)_2_ as compared to cells not preincubated with the hormone. The same differences were found when determining the mean intensity fluorescence (MIF) [(medium 197.5 ± 22.4; PRL 185.3 ± 11.68; anti-IgM F(ab′)_2_  342.2 ± 35.9; PRL 1 hour and anti-IgM F(ab′)_2_  292.3 ± 11.4)] (Figure 6).

**Figure 7:**
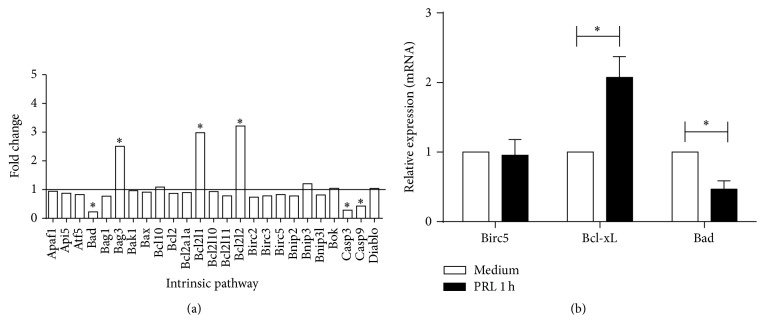
Expression of apoptotic genes modulated by PLR. WEHI-231 cells were incubated for 1 hour with PRL, and the expression of apoptosis genes was determined by (a) PCR array (mouse apoptosis) and (b) real-time PCR. ^*∗*^ *p* < 0.01.

The expression of genes involved in apoptosis in WEHI-231 cells treated with PRL for 1 hour was determined by a PCR array, finding that PRL modulates the expression of several members of the Bcl2 family, suggesting that PRL specifically targets the intrinsic pathway of apoptosis. In this family, PRL decreased the expression of the proapoptotic* Bad* (0.22) gene and increased the expression of antiapoptotic genes* Bag3* (2.50),* Bcl2l1* (2.98), and* Bcl2l2* (3.22), besides decreasing the expression of* Casp3* (0.29) and* Casp9 *(0.43) as shown in Figure 7(a) and in Supplementary Figure 3. The relative expression of some of these genes was confirmed by real-time PCR. PRL significantly increased the relative expression of* Bcl-xL* (2.07 ± 0.30) and decreased the expression of* Bad* (0.47 ± 0.12) as compared to cells incubated with medium alone (*p* < 0.01). No change was observed in the* Birc5* gene (0.95 ± 0.23) (Figure 7(b)).

### 3.4. PRL Affects Viability and Apoptosis of Immature B-Cells from MRL/lpr Mice

**Figure 8:**
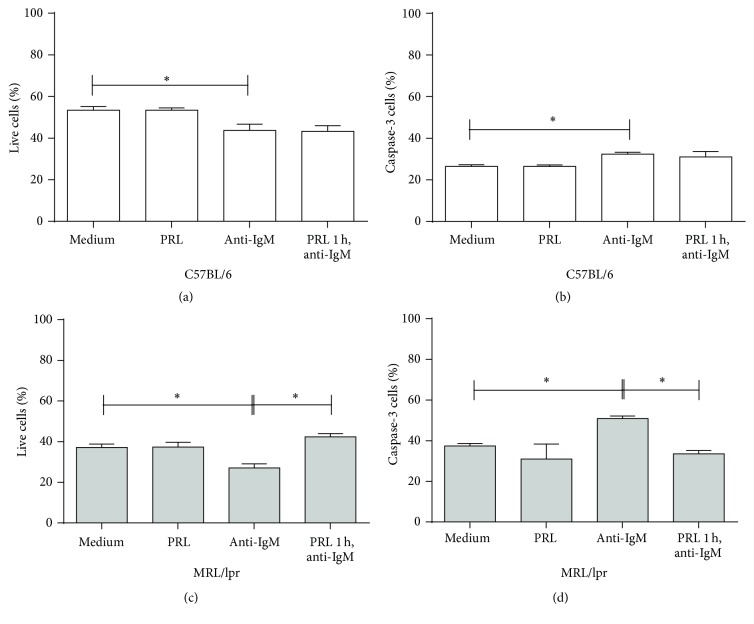
Effect of PRL on the viability and apoptosis of immature B-cells. Sorted immature B-cells from C57BL/6 or MRL/lpr mice were preincubated for 1 hour with PRL and incubated with anti-IgM F(ab′)_2_ antibody for 18 hours. The cells were stained with Ghost-Red and active caspase-3-FITC to determine apoptosis. (a) Percentage of live cells in C57BL/6 mice. (b) Percentage of caspase-3+ cells in C57BL/6 mice. (c) Percentage of live cells in MRL/lpr mice. (d) Percentage of caspase-3+ cells in MRL/lpr mice. ^*∗*^ *p* < 0.01.

To measure the effect of PRL on the viability and apoptosis of sorted immature B-cells from MRL/lpr and C57BL6 mice, Ghost-Red was used to measure viability, and active caspase-3 was used to measure apoptosis. In C57BL/6 control mice, a slight but statistically significant decrease (*p* < 0.01) in the viability of immature B-cells was observed when cells were incubated with anti-IgM F(ab′)_2_ (43.68 ± 3.01%) as compared to cells incubated with medium (53.42 ± 1.75%) or PRL (53.40 ± 1.14%). However, no difference in the viability was observed in cells preincubated with PRL and incubated with anti-IgM F(ab′)_2_ (43.22 ± 2.79%) as compared to cells not preincubated with the hormone (*p* = 0.7864) (Figure 8(a)). On the contrary, more profound changes were observed in the MRL/lpr immature B-cells; in cells incubated with anti-IgM F(ab′)_2_ for 18 hours (25.40 ± 1.27%), the percentage of live cells significantly decreased (*p* < 0.01) as compared to cells incubated with medium (37.96 ± 0.50%) or PRL (37.30 ± 2.43%). Moreover, cells preincubated with PRL and incubated with anti-IgM F(ab′)_2_ showed a statistically significant increase in the percentage of live cells (41.10 ± 2.26%) as compared to cells not preincubated with PRL (*p* < 0.01) (Figure 8(c)).

A similar result was obtained when addressing apoptosis. The percentage of cells with active caspase-3 significantly increased (32.40 ± 0.94%; *p* < 0.01) for immature B-cells from C57BL/6 mice that were incubated with anti-IgM F(ab′)_2_ as compared to those incubated with medium (26.43 ± 0.87) or PRL (26.47 ± 0.70%). However, no statistically significant difference (*p* = 0.2497) was found in the percentage of apoptotic cells when these cells were preincubated with PRL and incubated with anti-IgM F(ab′)_2_ (30.98 ± 2.61%) as compared to those not preincubated with PRL (Figure 8(b)). However, for MRL/lpr immature B-cells, the percentage of cells with active caspase-3 significantly increased (49.65 ± 0.64%; *p* < 0.01) for cells incubated with anti-IgM F(ab′)_2_ as compared to cells incubated with medium (37.80 ± 0.57%) or PRL (30.98 ± 7.39%). Moreover, the percentage of cells with active caspase-3 (34.75 ± 1.91) significantly decreased for cells preincubated with PRL and incubated with anti-IgM F(ab′)_2_ compared to cells not preincubated with PRL (Figure 8(d)).

## 4. Discussion

During the maturation of B-cells, elimination of autoreactive clones in bone marrow immature B-cells is a central control of tolerance, a mechanism that serves to avoid humoral self-responses [28–30]. Lack of elimination of B-cell clones with autoreactive BCRs favors the development of autoimmune diseases, such as SLE [3, 4]. In previous studies, we have demonstrated that, in mice that develop SLE, an increase in the serum levels of PRL decreases the absolute number of immature B-cells and increases transitional-1 cells in the spleen, correlating with the exacerbation of the disease [14, 23]. We consider that such observation could be explained by accelerated exit of bone marrow immature B-cells and increased arrival of B-cells to secondary lymphoid organs and that the tolerance mechanisms operating on immature B-cells could be compromised. Thus, the aim of this work was to determine whether PRL can rescue immature B-cells from apoptosis induced by the cross-linking of BCR. We first used the murine WEHl-231 cells, an* in vitro* model of immature cells widely used to study BCR mediated apoptosis, and then we corroborated the* in vitro* results using immature B-cells isolated from MRL/lpr mice, a mouse model of SLE-like disease.

Our results authenticated the notion that mouse WEHI-231 cells have the phenotype of immature B-cells. Moreover, these cells do not express genes that are exclusive of pro-B and pre-B-cells, such as IL7r, Rag1, and Igll1 [31, 32], but they express transcription factors that together with BCR signaling are critical for B-cell lineage commitment and maintenance [33–36]. Our results showed, for the first time, that this cell line expresses the PRL receptor similar to immature B-cells from C57BL/6, MRL, and MRL/lpr mice [14, 23]. In addition, the cells expressing the PRL receptor had better growth than those not expressing the PRL receptor when the cells were separated by the expression of the receptor (PRL receptor^+^ and PRL receptor^−^). This result may be attributed to the receptor potentially serving as a growth factor as reported in mouse B-cell hybridomas [37], or this result may be due to increased expression of antiapoptotic genes.

Different isoforms of the PRL receptor have been reported. In humans, the long isoform has been shown to be involved in the progression and metastasis of breast cancer, promoting the proliferation and viability of cancerous cells; the short isoform has been associated with antiproliferative and proapoptotic effects [38–40]. Our results indicate that WEHI-231 cells only express the mRNA for the long isoform of the PRL receptor. PRL modulates the expression of genes from the Bcl2 family that participate as part of the intrinsic pathway of apoptosis, which correlated with decreased apoptosis induced after cross-linking of the BCR. This provides for a potential mechanism of rescuing self-reactive clones from clonal deletion.

Our results and others indicate that PRL protects cells from apoptosis when challenged with different stimuli, an effect in which increasing the expression of antiapoptotic genes of the intrinsic pathway of apoptosis probably has a central role. Prolactin-treated spleen B-cells from B6.Sle3 mice were more resistant to apoptosis in [41]; PRL protected Nb2 cells from apoptosis mediated by dexamethasone through the expression of the Bcl-xL gene [42]; and in breast cancer cells, PRL increased the mRNA and protein expression of Bcl2 [43]. Other studies support the notion that the Jak/Stat signaling pathway modulates the expression of apoptotic genes from the Bcl2 family [44–46]. In an arthritis model, it has been discovered that the Jak2/Stat3 pathway activates the transcription of antiapoptotic genes, such as Bcl2, and rescues chondrocytes from apoptosis [47]. Our studies demonstrated that PRL increases the expression of* Stat5b* in WEHI-231 cells (Supplementary Figure 4). This suggests that the interaction of PRL with the long isoform of the receptor expressed by the immature B-cells signals through the Stat5b pathway, modulating several Bcl2 family members from the intrinsic pathway of apoptosis to rescue the cells from death. However, it is necessary to perform more experiments to determine the signaling pathway of the long isoform of the PRL receptor in immature B-cells.

In other autoimmune diseases, such as arthritis and multiple sclerosis, it has been described that PRL increases the expression of antiapoptotic genes, such as Bcl2, and decreases the expression of proapoptotic genes, such as Trp63 and Bax, suggesting that this hormone may favor the progression of the disease [47, 48]. Our results showed that PRL promotes the viability of immature B-cells that should be subjected to negative selection, rescuing them from apoptosis, both in a cell line and in a model of SLE (MRL/lpr). PRL may prevent apoptosis of bone marrow immature B-cell clones that recognize self-antigens (potentially autoreactive clones), which may allow maturation of autoreactive B-cell clones, thus increasing the risk of developing autoimmune diseases. These results, together with our previous observations in* in vivo *studies, indicate an important effect of PRL on B-cell maturation and the development of the disease.

## 5. Conclusions

WEHI-231 cells express the long isoform of the PRL receptor associated with induction of resistance to apoptosis. In these cells, PRL modulates the expression of genes from the intrinsic pathway of apoptosis increasing the relative expression of Bcl-xL (antiapoptotic gene) and decreases the expression of Bad (proapoptotic gene), which may prevent the apoptosis of these cells induced by the cross-linking of BCR. Furthermore, PRL increases the viability of immature B-cells by rescuing them from apoptosis (through BCR cross-linking) preferentially in cells from mice that developed SLE (MRL/lpr). These results suggest that PRL may favor the maturation of self-reactive clones, thus allowing the onset of autoimmune diseases.

## Supplementary Material

Supplementary 1. Purification of PRL receptor-positive WEHI-231 cells.
Supplementary 2. Viability of WEHI-231 cells PRL receptor+ and PRL receptor−.
Supplementary 3. Expression of apoptotic genes modulated by Prolactin.
Supplementary 4. Expression of Stat5b in WEHI-231 cells.
