# Supplementary material for: Prolactin Rescues Immature B-Cells from Apoptosis Induced by B-Cell Receptor Cross-Linking
Source: J Immunol Res. 2016 May 24;2016:3219017. doi: 10.1155/2016/3219017 (PMC4894992; doi:10.1155/2016/3219017)
Supplement: Supplementary file 1 — Supplementary 1. Purification of PRL receptor-positive WEHI-231 cells. Supplementary 2. Viability of WEHI-231 cells PRL receptor+ and PRL receptor−. Supplementary 3. Expression of apoptotic genes modulated by Prolactin. Supplementary 4. Expression of Stat5b in WEHI-231 cells. [file 3219017.f1.zip › description.docx]

Supplementary 1. Purification of PRL receptor-positive WEHI-231 cells

WEHI-231 cells were stained with the following antibodies: IgM, CD19, and PRL receptor 20min, 4oC in darkness. Ghost-Red was used to marker dead cells 30min, 4oC in darkness. The cells were purified by sorting in FACSAria cytometer from BD using the following selection markers: Ghost-Red-, IgM+, CD19+, and PRL receptor+. The purity of the collected populations varied between 95% and 98%.

Supplementary 2. Viability of WEHI-231 cells PRL receptor+ and PRL receptor-

The WEHI-231 cells PRL receptor+ and PRL receptor- obtained by sort were maintained in culture with RPMI 10% FBS at 37°C in 5% CO2; viability was determined by labeling with Ghost-Red 30min, 4oC in darkness. Dead cells were Ghost-Red+ and the live cells were Ghost-Red-.

Supplementary 3. Expression of apoptotic genes modulated by Prolactin

The WEHI-231 cells PRL receptor+ were incubated for 1 hour with PRL; RNA was extracted and cDNA was obtained to determine the expression of apoptosis genes by PCR array using Mouse PAMM-012Z RT² Profiler™ PCR Array a) Extrinsic Pathway Apoptosis genes and b) Other genes of apoptosis. *p<0.01

Supplementary 4. Expression of Stat5b in WEHI-231 cells

The WEHI-231 cells PRL receptor+ were incubated for 1 hour with PRL; RNA was extracted and cDNA was obtained to determine the relative expression of the Stat5b gene by PCR array using Mouse CAPM12814 RT² Profiler™ PCR Array.
