# Supplementary figures and images for: Prolactin Rescues Immature B-Cells from Apoptosis Induced by B-Cell Receptor Cross-Linking
Source: J Immunol Res. 2016 May 24;2016:3219017. doi: 10.1155/2016/3219017 (PMC4894992; doi:10.1155/2016/3219017)

Pre-Sort

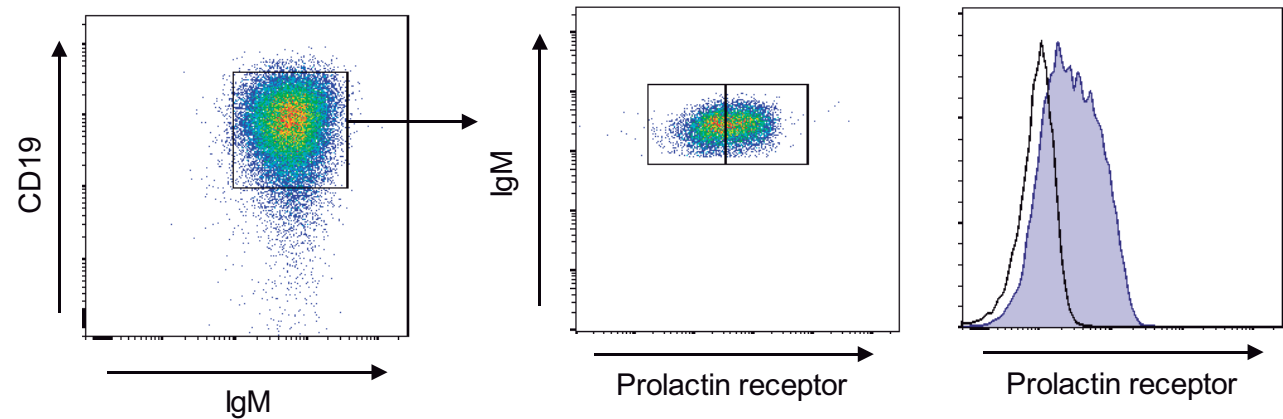

Post-sort

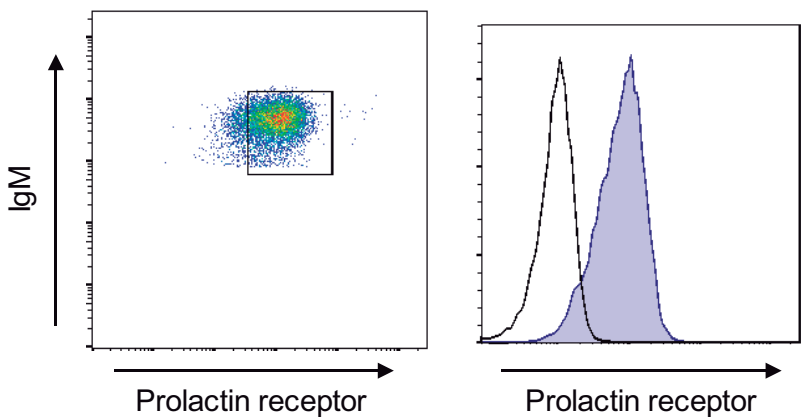

Supplement: Supplementary file 1 — Supplementary 1. Purification of PRL receptor-positive WEHI-231 cells. Supplementary 2. Viability of WEHI-231 cells PRL receptor+ and PRL receptor−. Supplementary 3. Expression of apoptotic genes modulated by Prolactin. Supplementary 4. Expression of Stat5b in WEHI-231 cells. [file 3219017.f1.zip › Suplementary 1.pdf]

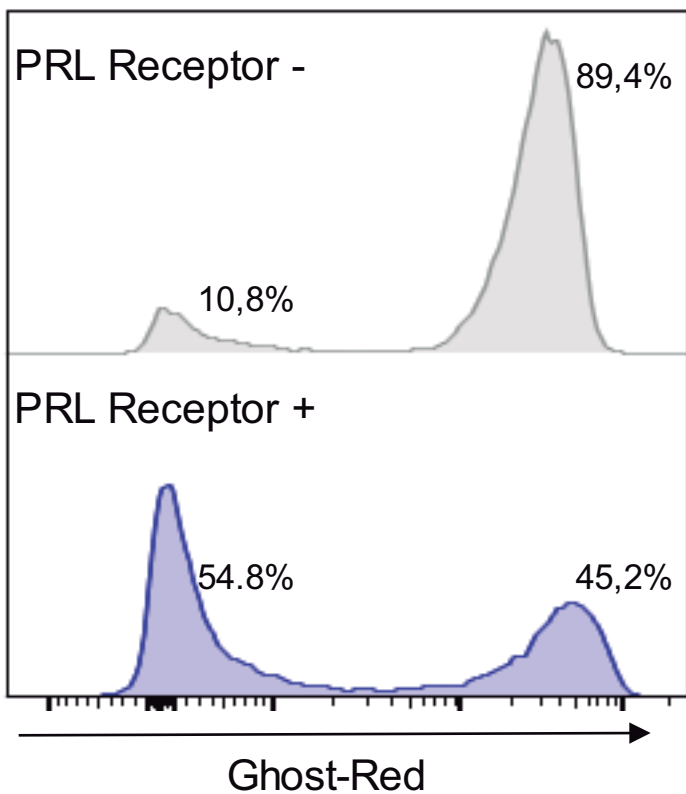

Supplement: Supplementary file 1 — Supplementary 1. Purification of PRL receptor-positive WEHI-231 cells. Supplementary 2. Viability of WEHI-231 cells PRL receptor+ and PRL receptor−. Supplementary 3. Expression of apoptotic genes modulated by Prolactin. Supplementary 4. Expression of Stat5b in WEHI-231 cells. [file 3219017.f1.zip › Suplementary 2.pdf]

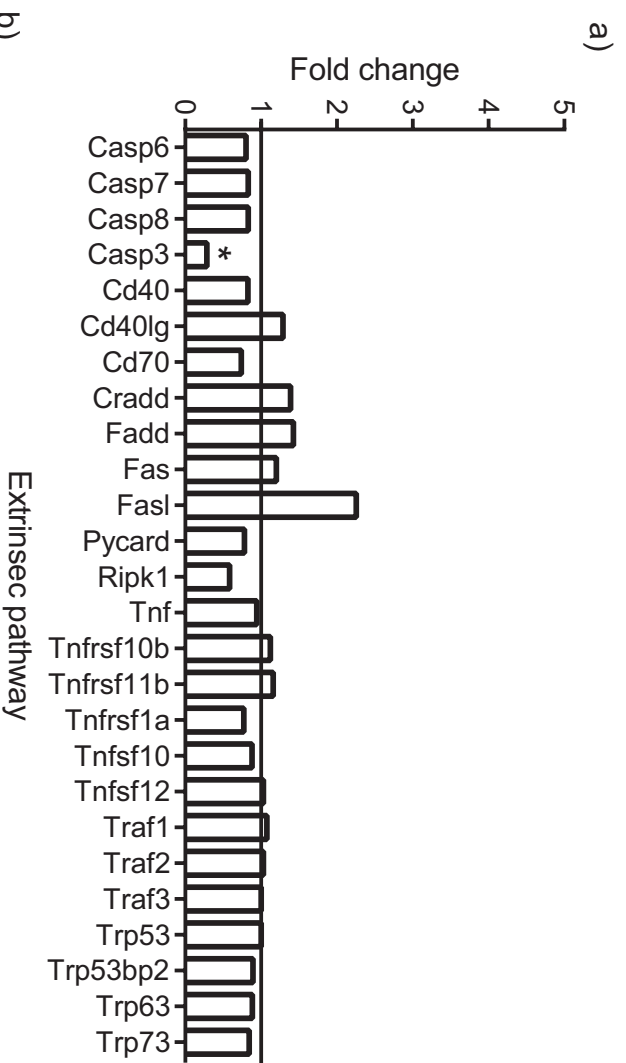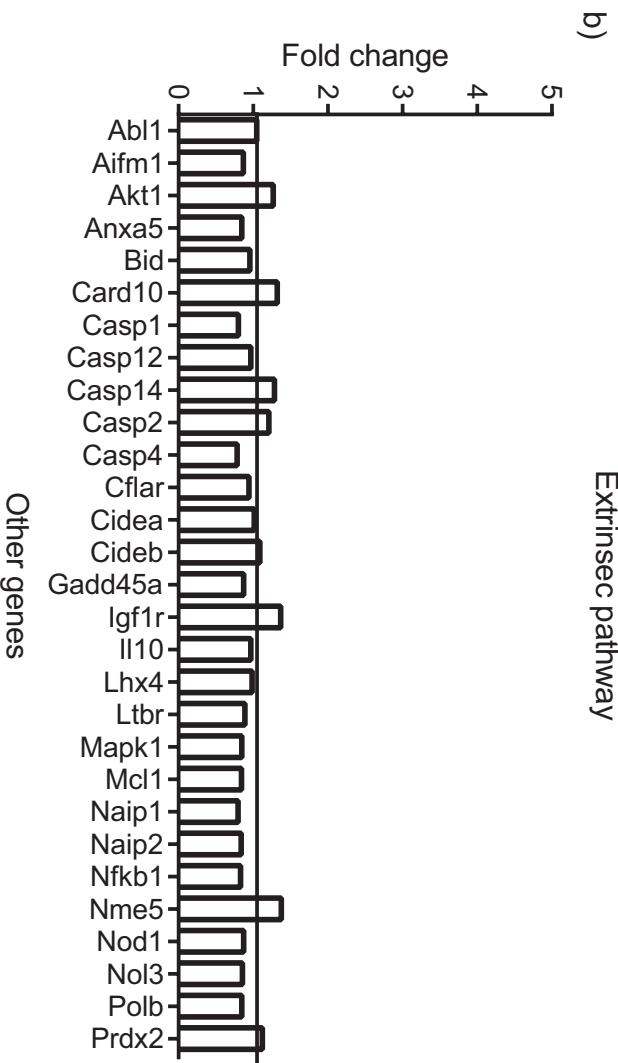

Supplement: Supplementary file 1 — Supplementary 1. Purification of PRL receptor-positive WEHI-231 cells. Supplementary 2. Viability of WEHI-231 cells PRL receptor+ and PRL receptor−. Supplementary 3. Expression of apoptotic genes modulated by Prolactin. Supplementary 4. Expression of Stat5b in WEHI-231 cells. [file 3219017.f1.zip › suplementary 3.pdf]

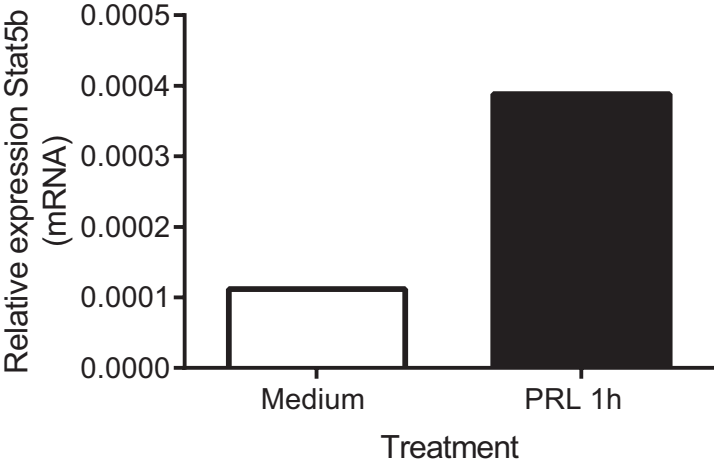

Supplement: Supplementary file 1 — Supplementary 1. Purification of PRL receptor-positive WEHI-231 cells. Supplementary 2. Viability of WEHI-231 cells PRL receptor+ and PRL receptor−. Supplementary 3. Expression of apoptotic genes modulated by Prolactin. Supplementary 4. Expression of Stat5b in WEHI-231 cells. [file 3219017.f1.zip › Supplementary 4.pdf]
